# The history of the neglected tropical disease movement

**DOI:** 10.1093/trstmh/trab015

**Published:** 2021-01-28

**Authors:** David H Molyneux, Anarfi Asamoa-Bah, Alan Fenwick, Lorenzo Savioli, Peter Hotez

**Affiliations:** Department of Tropical Disease Biology, Liverpool School of Tropical Medicine, Pembroke Place, Liverpool, L3 5QA, UK; University of Ghana Medical Centre, Legon Accra, Ghana; School of Public Health Imperial College Norfolk Place W2 1PG, UK; P.O. Box 267, Chake Chake, Pemba Island, Zanzibar, United Republic of Tanzania; National School of Tropical Medicine, Baylor College of Medicine, Houston, TX 77030, USA

**Keywords:** millennium development goals, neglected tropical diseases, partnerships, sustainable development goals

## Abstract

The history of the neglected tropical disease movement is seen through the lens of authors who worked during the last 4 decades in different roles and in different settings, from Western-based laboratories to clinical roles in endemic countries and in critical policy roles in the World Health Organization (WHO). The authors seek to identify key players from the introduction of the word ‘neglected’ by the late Kenneth Warren in his Rockefeller Foundation–supported Great Neglected Diseases of Mankind movement through to the more recent developments after the London Declaration of 2012. The role of the various actors—endemic countries, major pharmaceutical companies, the WHO, non-government development organizations, bilateral donors and academia—are discussed. The critical events and decisions are highlighted that were essential enabling factors in creating a viable and successful movement and with a resultant massive global public health and antipoverty impact. The importance of advocacy is emphasized in creating the momentum to establish a globally recognized public health ‘brand’ as a target in the United Nations Sustainable Development Goals.

## Introduction: 20th century origins

Estimates suggest that approximately 1 in 10 of the world's population live in extreme poverty (on <$1.90/d), with most infected with one or more of the neglected tropical diseases (NTDs). Indeed, the term ‘neglected tropical diseases’ describes a transition from the 19th century study of tropical diseases to one that prioritized the plight of neglected populations living in extreme poverty.^1^ During the late 1970s, Kenneth S. Warren, Head of Health at the Rockefeller Foundation, launched a network of research laboratories devoted to the study of parasitic diseases. He branded them as ‘great neglected diseases (GND) of mankind’ to incentivize scientists who previously worked on model organisms to apply their knowledge to infectious diseases of the poor. Warren recognized how diseases such as schistosomiasis and malaria were ignored by the evolving disciplines of immunology and molecular biology.^2–4^ He was successful in making a humanitarian appeal to these scientists. For example, Warren pointed out how schistosomiasis caused liver disease in 100 million people and yet the total research expenditures worldwide for this disease were <$5 million^3^ and schistosomiasis was not prioritized by the community committed to the study of hepatic disease. Many scientists began their basic research through Warren's GND Network,^5,6^ recognizing the humanitarian benefits of coming together; this led to the grouping of these conditions under the moniker of ‘neglected’.

In parallel with Warren's initiative in 1976, the World Health Organization (WHO) Special Programme for Research and Training in Tropical Diseases (TDR) was established under the auspices of the World Bank and United Nations Development Programme (UNDP), a partnership of multilateral and bilateral donors and disease-endemic countries to study a similar portfolio of diseases under the successive leadership of Howard Goodman, Adetokundu Lucas and Tore Godal.

In Africa, the British government and the European schools of tropical medicine were also supporting research institutions in endemic areas, but there was an equal urgency to simultaneously prioritize low-cost interventions with existing medicines. Solving tropical disease dilemmas required strategies that would be effective and yet inexpensive. A hint of things to come were initial efforts to promote mass treatment and worm control with an anthelminthic drug. The concept was pioneered by Frank Hawking during the 1950s and 1960s using diethylcarbamazine citrate (DEC) for lymphatic filariasis (LF)^7,8^ and by the Chinese using DEC-fortified salt for filariasis control in a population of 350 million people.^9^ It took some 50 y for this concept to be accepted and implemented globally.

## Preventive anthelminthic chemotherapy: mass treatment without individual diagnosis

Despite the efforts highlighted above, by the 1990s there remained a dearth of funding for both research and treatment of diseases of poor populations and therefore no significant expansion in the number of scientists interested or able to work on these conditions. By the turn of the millennium, the study of infectious tropical pathogens had fallen behind other areas of the biomedical sciences. Parasites were not easily amenable to modern molecular and immunological methods or rapidly accelerating advances in transmission dynamics and mathematical modeling.^6,10,11^ Then two important developments occurred.

The first major advance in the diseases of the poor was the entry of multinational pharmaceutical companies, which began turning their attention to the treatment of global tropical infections. Early results from TDR-sponsored studies on leprosy resulted in the development of multidrug therapy (MDT; rifampicin, clofazimine and dapsone), donated by Novartis (then Ciba-Geigy) in 2000 following approval of MDT in 1984 by the WHO. Increasingly it became recognized that multinational pharmaceutical companies might play a significant humanitarian role in the mass treatment of global human parasitic infections. A pioneer was Nobel Laureate William Campbell, who led efforts at MSD to develop and test anthelminthic drugs of the avermectin class following their initial discovery and isolation from *Streptomyces* bacteria by Satoshi Ōmura and colleagues in Japan.^12^ Initial work focused on veterinary applications to treat livestock for intestinal helminth and ectoparasite infections. Soon clinical trials confirmed the therapeutic effect of the drug ivermectin in treating human onchocerciasis (river blindness),^13^ prompting MSD Chief Executive Officer Roy Vagelos to announce the donation of Mectizan (ivermectin) ‘for as long as needed’ through the Mectizan Donation Program.^14^ Both Mectizan and MDT donations shaped a new paradigm in which a drug discovered or developed by a major pharmaceutical company might be redirected to treat human diseases in global programs of mass drug administration.

The use of Mectizan became instrumental for the success of the World Bank's Onchocerciasis Control Programme (OCP) in West Africa in its later stages when it was employed to supplement vector control to reduce microfilarial loads.^15^ The Mectizan Donation Program was an enabling factor when the World Bank created the African Programme for Onchocerciasis Control (APOC) in 1995, allowing expansion of onchocerciasis programmes based on the concept of community-directed treatment with ivermectin (CDTi), an approach developed by the WHO/TDR, OCP and APOC-sponsored research in all of the remaining onchocerciasis-endemic countries in Africa.^16^ The initial objective of the APOC was to create sustainable Mectizan delivery systems using CDTi within 5 y.^16^ In addition, the multidonor financing of the APOC was supplemented by the strong commitment of a number of non-government development organizations (NGDOs) who assisted in the implementation of country programmes while also providing some 25% of the financing.^17^

At the same time, a programme for the control of onchocerciasis in the six endemic countries in the Americas (Onchocerciasis Elimination Programme in the Americas [OEPA]) was launched based on Mectizan treatment alone given twice yearly.

A second accelerant was the theoretical framework of population and transmission dynamics models of human helminth infections shaped by Anderson and May^10^ and Schad and Anderson,^11^ with evidence that proof-of-concept of such constructs could be successfully applied to the deworming of children with intestinal helminths.

Such efforts prompted the initial donation of mebendazole, by Johnson & Johnson, and later albendazole by GlaxoSmithKline.^18^ In parallel, Davis and Wegner^19^ showed the potential impact of praziquantel on human schistosomiasis, and some 25 y later the Schistosomiasis Control Initiative, seed funded by the Bill & Melinda Gates Foundation (BMGF), was able to scale up the use of praziquantel, eventually leading to a donation of the drug by Merck KGaA. A major result in 2007, through efforts by the WHO and the Financial Times, was that Merck KGaA signed an agreement for the donation of praziquantel for schistosomiasis for school-age children. Initially 20 million tablets per year were donated, but by 2017 the donation had reached 250 million tablets.

Meanwhile, Mabey et al.^20^ led efforts to examine mass treatment of trachoma with azithromycin, leading to a donation of that drug by Pfizer through the International Trachoma Initiative (ITI) founded by Joseph Cook, which also increased to >120 million doses annually by 2015. In 2000, the Global Programme to Eliminate Lymphatic Filariasis (GPELF) was launched following a World Assembly Resolution in 1997 calling for elimination of the disease as a public health problem. Combinations of either albendazole and diethylcarbamazine (in Asia) or albendazole and ivermectin (in Africa in countries endemic for onchocerciasis) were then employed to treat millions of people annually to protect them from LF following studies carried out through funding from the TDR.^21^

## NTDs: a pro-poor framework for the new millennium

Reaching the poorest populations and treating and preventing disabling infections became an aspirational advocacy strategy. Decision makers began to recognize that implementation of mass treatment programmes could promote economic development. The economist Jeffrey Sachs proposed linking Africa's development to the control of malaria and other diseases^22,23^ and chaired a Commission on Macroeconomics and Health.^24,25^ The commission report was a landmark document linking global health to poverty, also explaining how diseases such as malaria and human immunodeficiency virus (HIV)/acquired immune deficiency syndrome (AIDS) trapped populations living in low- and middle-income countries in a cycle of poverty.^24,25^ It also helped to spark the elevation of infectious disease control to global policy makers, leading to its inclusion as Millennium Development Goal (MDG) 6: ‘To combat AIDS, malaria, and other diseases’. Accordingly, Sachs, as adviser to the United Nations (UN) Secretary-General, initiated the formation of the Global Fund to Fight AIDS, Tuberculosis and Malaria as a financial instrument to support countries in controlling these so-called big three diseases. However, it also became apparent that the Global Fund portfolio excluded some of the important neglected diseases highlighted above.

The MDG 6 focused on HIV/AIDS and malaria at the expense of ‘other diseases’, including polio. Relegating parasitic infections such as schistosomiasis, hookworm and LF to the ‘other diseases’ essentially left more than a billion people unserved and unrepresented. In response, individual scientists began advocating how particular diseases, such as LF, onchocerciasis, schistosomiasis, trachoma and hookworm infection deserved recognition as poverty-causing conditions.^26,27^ Neglected disease advocates further emphasized how interventions for these parasitic diseases, especially through mass drug administration^28^ or new vaccines^29^ would enhance development. Fortunately, coinciding with the launch of the MDGs, the new BMGF began the first of some of its major global health contributions to NTDs by supporting academic institutions and NGDOs not only for research on the basic science of NTDs but, especially importantly, for operational research to ensure that the specific, real-world challenges of NTD programmes were recognized and met (e.g. where the diseases are found, how to identify and treat entire at-risk populations, how to ensure population compliance, how to define and meet target thresholds, what diagnostic tools and sampling strategies are required and available, etc.). Without answers to such questions, workable programmes cannot be developed, and without workable programmes, long-term support cannot be sustained. The BMGF also committed support for implementation of the OEPA and the Guinea Worm Eradication Programme through the Carter Center.

A major turning point in global interest in tropical diseases was the appointment in 2003 of J. W. Lee as Director General of the WHO. Lee had experience working in leprosy in the Republic of Korea as a young medical officer. At the WHO as Director General, Lee restructured the communicable diseases area as one of the top priorities together with the ‘3 by 5’ AIDS treatment campaign. In 2003, Hiroyoshi Endo was charged with reorganizing this area of work, leading to renewed interest by the WHO's member states, donors, the pharmaceutical industry, academia and world experts.

As a result of meetings in Berlin organized by the WHO and the Gesellschaft für Technische Zusammenarbeit (GTZ) in 2003 and 2005, the current framework of the NTDs began to take shape. In a 2004 Lancet paper,^30^ Molyneux pointed out that huge successes had already been achieved in combatting several tropical infections, listing filariasis in China, the OCP in West Africa, Chagas’ disease in the Southern Cone countries of the Americas via indoor residual spraying, leprosy with MDT and progress in Guinea worm eradication initiated through the advocacy of former-President Jimmy Carter in the late 1980s during the International Water Decade. The major tenets of the concept of the NTDs were then detailed in two back-to-back papers published in the open access journal PLoS Medicine in 2005 and 2006.^31,32^ These became the first two papers indexed in PubMed specifically referring to NTDs, reporting on major features that included the following observations:

- There were approximately 13 diseases of the poor that could be branded as NTDs due to their high prevalence and pervasiveness among populations living in extreme poverty.
- These diseases geographically overlapped and were co-endemic such that impoverished populations were polyparasitized.
- The effects of polyparasitism translated into lost productivity and disability (often irreversible), with consequential socio-economic decline.
- At least 7 of the 13 diseases could be targeted simultaneously in an inexpensive ‘rapid impact’ package of medicines donated by pharmaceutical companies or available as low-cost generics.

A further consideration was how NTDs also complicated the treatment of malaria and HIV/AIDS through synergistic and additive effects operating through anaemia and altered host immune responses. Examples included the combination of hookworm, schistosomiasis and malaria in pregnancy or the role of female genital schistosomiasis in promoting susceptibility to HIV/AIDS.^33^

## Taking action: translating science into policy and practice

Efforts to redirect the ‘other diseases’ of the MDGs towards an NTD ‘brand’ and offer low-cost solutions were mostly led by academic scientists working with the WHO.^34^ At that point there was no roadmap for transitioning the published scientific articles in biomedical journals into global action. However, in the absence of an established advocacy network, scientists working on the NTDs recognized a new reality that they needed to lead such efforts and spearhead implementation. They created a unique dynamic in which scientists began working directly with leaders of the US Congress and the UK Parliament to allocate funding to support the scale-up of rapid impact packages of donated medications targeting NTDs.^31–34^ The WHO responded by forming a new Department of NTDs following the 2005 Berlin meeting. Over this period, scientific findings rapidly transitioned into global health policy on several fronts: in the USA and UK, the WHO and its regional offices, increased pharmaceutical company interest, commitment from NGDOs and the existing disease-specific partnerships. The focus was a package of simple treatments given annually that could target seven major NTDs: the three soil-transmitted helminthiases, LF, onchocerciasis, schistosomiasis and trachoma. Because of drug donations from the pharmaceutical companies, the package could be administered for <$1 per person and often for <$0.40.^32,33,35,36^ These medicines included albendazole, mebendazole, praziquantel, DEC, ivermectin and azithromycin. It became increasingly apparent to global policymakers that this approach represented one of the most cost-effective ‘best buys’ in global public health.^37^ In addition, Sanofi-Aventis committed to donate therapies for human African trypanosomiasis and leishmaniasis and Gilead donated its new product ambisome for visceral leishmaniasis.

The next step was to mobilize resources to deliver these medicines and support the health ministries in disease-endemic nations. A critical impact was the decision by the G8 in Hokkaido (Japan) in 2008 to help to control and eliminate several NTDs. This was initiated by the personal commitment of Prime Minister Hashimoto of Japan, advised by some committed parasitologists led by the late Tsutomu Takeuchi. It was initially raised as an agenda item at a G8 Summit in Denver, CO, USA in 1997 and was endorsed at the Birmingham (UK) Summit in 1998 to tackle the issue globally. The Global Parasite Control for the 21st Century initiative, better known as the Hashimoto Initiative, was a critical visionary initiative not immediately endorsed by many G8 states. However, it did stimulate increased advocacy efforts in several G8 countries following the decision in Hokkaido 10 y later. Initially efforts were focused on the US and UK governments, and later private sources of funding. In the USA, Peter Hotez and Eric Ottesen worked through the Global Health Council (under the direction of Niels Dulaire) to explain to the Bush White House and key congressional leaders about the highly cost-effective opportunity to control NTDs. They persuaded Congress in 2005 to provide initial financial support to programmes targeting NTDs by taking advantage of the donated medicines available. In 2006, the US Agency for International Development (USAID) established an NTD programme for this purpose, allocating $100 million over 5 y to operate through contractors, NGDOs and the ministries of health in disease-endemic countries.

In 2005, Hotez worked with Gavin Yamey from the Public Library of Science (PLoS) to secure seed support from the BMGF and create PLoS Neglected Tropical Diseases, the first open access journal for NTDs,^38^ in addition to writing a book entitled, Forgotten People Forgotten Diseases.^39^ The BMGF also supported advocacy and resource mobilization for NTDs by establishing a Global Network for NTDs, initially based in Washington, DC,^33,40^ prior to transitioning it to Uniting to Combat NTDs in London, together with an END (End Neglected Diseases) Fund based in New York. Following the transition from the Bush to the Obama administration, Ezekiel Emanuel and Office of Management and Budget Director Jack Lew and others helped to maintain enthusiastic support for NTDs. A further dimension towards advancing pro-poor policies was the realization that NTDs are also widespread among the poor living in the USA^41^ and the G20 group of nations, a concept referred to as ‘blue marble health’ to differentiate it from traditional global health norms of developed versus developing countries.^41,42^

In the UK, Alan Fenwick was able to work with the team writing the Commission for Africa Report launched by Prime Minister Tony Blair at the Gleneagles G8 Summit in 2005 to obtain recognition for the need to address NTDs. Ultimately, through the UK Department for International Development (DFID), the UK expanded initial support from USAID and the BMGF. Funds from these organizations helped to either create or sustain the Schistosomiasis Control Initiative (SCI, founded by Alan Fenwick and initially based at Imperial College, London) and the Lymphatic Filariasis Programme based at the Liverpool School of Tropical Medicine under David Molyneux and funded by the DFID in partnership with GlaxoSmithKline. Molyneux and Fenwick were then asked in 2008 to prepare suggestions for how UK funding could be best deployed to support NTD programmes, leading to an initial £50 million commitment that was subsequently increased to an additional £195 million (announced at the meeting in London in January 2012). After 2010, a key individual was Sir Stephen O'Brien, as a minister in the DFID (later UN Undersecretary General for Humanitarian Affairs), who recognized the cogent arguments from NTD advocates for the health and development objective as an exemplar of effective partnerships and the value of money provided by donor support. His initiative led to an important advocacy forum being established in the UK parliamentary system ‘The All-Party Committee on Malaria and NTDs’, which continues to meet regularly.

The recognition that some NTDs that caused significant mortality, notably the leishmaniases and trypanosomiases, required medical supervision for treatment and depended on drugs that were expensive, toxic to patients and involved long courses of treatment led to the establishment of the Drugs for Neglected Disease Initiative (DNDi), a product development partnership with the objective of developing more effective and safer drugs initially for the leishmaniases and trypanosomiases, but which has now expanded to include conditions such as mycetoma and a macrofilaricide for onchocerciasis and filariasis. Later, the Foundation for Innovative New Diagnostics (FIND) was created as a product development partnership (PDP) for diagnostics, as were several PDPs for ‘antipoverty’ vaccines, including the Infectious Disease Research Institute (IDRI, founded by Steve Reed), Human Hookworm Vaccine Initiative (HHVI, founded by Peter Hotez), which later became the Texas Children's Center for Vaccine Development, and the International Vaccine Institute (IVI).^1^

In Geneva, following the 2005 Berlin meeting at the WHO, J. W. Lee and Anarfi Asamoa-Baah worked with Lorenzo Savioli to establish an inaugural Department of NTDs to provide technical support for integrated mass drug administration, later renamed preventive chemotherapy or preventive treatment, and diseases that required more intensive clinical management. Initially the WHO adopted the 13 diseases but subsequently expanded this list to 20 conditions.^43^ A turning point in the efforts against these diseases was achieved after the first Global Partner's Meeting convened by the WHO in 2007, an initiative outside any formally structured partnership that resulted in a shared commitment to support the WHO's strategies, goals and targets.^44^ Key to the political support by all partners was the endorsement by the WHO member states of a series of specific resolutions for each NTD in the WHO portfolio to ensure political backing for disease prevention, control, elimination and eradication strategies. The capstone of this essential work was the approval in 2013 of an overarching comprehensive resolution on NTDs (WHA 66.12, World Health Assembly [WHA] Resolutions on Neglected Tropical Diseases: 1948–2019, available from: https://www.who.int/neglected_diseases/mediacentre/resolutions/en/) that ensured the full backing of the WHO's governing bodies of all the key public health strategies developed by the NTD community for >40 y.

## The London Declaration

As the establishment of the NTDs as a credible ‘brand’ developed,^31–33^ the NGDOs became increasingly important players in support of country implementation. Beyond SCI and the LF programmes, Sightsavers, UK, began supporting wider issues of disability where previously it had focused on the visually impaired. Large NGDOs such as Helen Keller International (HKI), Christoffel-Blindenmission (CBM), the Carter Center, the Task Force for Global Health and the leprosy NGDOs, through the International Federation of Anti-Leprosy Associations, joined the NTD advocacy movement, as did several large USAID contractors such as RTI International and FHI360, eventually leading to the creation of a more formally structured partnership, the NTD Network (NNN), now embracing some 90 organizations.^44^ In addition, the establishment of the Coalition for Operational Research for NTDs, in the Task Force for Global Health, coordinated the drive to address the operational research questions and ensure the research community focussed on programmatically relevant questions tied to achieving the 2020 goals was also a pivotal event. It also linked three key donors—the BMGF, USAID and DFID—in their commitment to support and improve the scale-up of programmes of new interventions while ensuring improved donor coordination.

The years 2002–2012 were a success for the advocacy and implementation of preventive treatment with the drugs praziquantel (against schistosomiasis), ivermectin (against river blindness), ivermectin and albendazole in Africa (against LF), DEC and albendazole (against LF outside of Africa), azithromycin (against trachoma) and mebendazole and albendazole (against intestinal helminths). The numbers reached by organizations assisting ministries of health and education in the endemic countries increased as more medications were donated by pharmaceutical companies.

In January 2012, a meeting in London, chaired by Bill Gates, was convened to bring together the main players in the support of NTDs: the WHO, the BMGF, the USA and UK as bilateral donors, NGDOs and the pharmaceutical industry. The meeting and resulting London Declaration established not only increased commitment, but also Uniting to Combat NTDs as an advocacy group with the mandate to expand the partnerships and draw in new partners and donors (www.unitingtocombatntds.org). By 2015, a critical milestone was reached, when the numbers of annual treatments reached more than one billion people for NTDs, primarily with donated essential medicines, often in a package of interventions as first outlined in 2005. Today it is worth noting that much of the leadership of this system of organizations committed to NTDs has been and continues to be shaped by charismatic women. The visionary leadership of Uche Amazigo, Director of the APOC, who passionately articulated the personal stories of onchocerciasis patients; Julie Jacobson and Katey Owen of the BMGF; Mwele Malecela, who directed the Tanzania LF programme and is now the director of the WHO NTD Department; Lisa Rotondo, who heads the RTI NTD programme; Maria Rebollo Pollo, who is responsible for the Expanded Special Project for Elimination of NTDs (ESPEN) of WHO/AFRO; Wendy Harrison, who leads the Schistosomiasis Control Initiative Foundation; Thoko Elphick-Pooley, of Uniting to Combat NTDs; Ellen Agler, chief executive officer of the END Fund; Emily Wainwright, at USAID; Delna Ghandi of DFID and Caroline Harper of Sightsavers.

Following progress in the goals and targets of the 2012 WHO roadmap that inspired the London Declaration and the scale-up several new key themes began to emerge. The major successes achieved by endemic countries in achieving elimination of some NTDs stimulated increased interest and justified investment in programmes building a firm evidence-based platform for continued support. In addition, there is the realization that NTDs disproportionately affect girls and women living in poverty, perhaps best illustrated by the plight of some 40 million African adolescent girls and young women affected by female genital schistosomiasis.^44^ Despite an abundance of evidence that female genital schistosomiasis increases the chance of HIV/AIDS infection, it remains difficult to persuade global policymakers to link programs for these conditions. Thus a tragic firewall remains separating praziquantel for schistosomiasis from the US President's Emergency Plan for AIDS Relief and the Global Fund. Yet another concern is the increasing awareness of how NTDs affect mental health through both direct effects on the central nervous system and through their stigmatizing and psychological effects.^45,46^ NTD control has the potential to greatly reduce the global burden of mental illness.

Finally, there is the recent finding that as mass treatment expands and has reached more than one billion people annually we are beginning to see important but unexpected collateral benefits of these programs, leading to the control and elimination of NTDs such as yaws and scabies (later added to the WHO's portfolio of NTDs ), as well as overall reductions in child morbidity and mortality through mechanisms not yet determined.^47^ By the end of the 2010s there was growing awareness that we might see both the control and even elimination of some key NTDs as well as improve the overall health of girls and women, and young children, while making a significant impact on global mental health.

## Future directions

There was a sense of optimism that substantial progress had been made towards the elimination of several NTDs, including LF, onchocerciasis and trachoma, while promising progress in countering the scourge of skin NTDs, notably scabies and yaws, has been achieved. The numbers of new cases are at an historic low and a new oral treatment, fexinidazole, has been approved for use while new and exciting technologies for diagnostics and monitoring are in the development pipeline. New efforts to document the impact of these activities on reducing poverty levels were under way. The number of people living in extreme poverty has fallen dramatically since 2000, to the point where <750 million people lived below the World Bank poverty level of $1.90/d. For a period of 5 consecutive years, until the end of 2019, >1 billion people had received NTD treatments.

However, in January 2020, coronavirus disease 2019 (COVID-19) emerged from China, becoming a global pandemic. Indeed, the case has been made that COVID-19 itself is a health disparity and an NTD.^48^ The COVID-19 pandemic of 2020 has disrupted programmes of mass drug administration and other NTD control measures.^49^ Alternative approaches for ensuring access to preventive chemotherapy have been developed by the WHO in concert with member states, implementing agencies, the pharmaceutical industry and NGDOs developing a ‘hybrid’ approach.^50^ There is a serious risk NTDs will rebound in areas where effective control or elimination had occurred pre-COVID-19. The re-emergence of NTDs in some settings, be it due directly to COVID-19, to health system interruptions from war and political collapse,^51^ from demographic change such as urbanization in African and Asian megacities^52,53^ or climate change,^54^ can be predicted. The NTD community has engaged with the WHO in developing the new WHO/NTD Road Map 2021–2030, now endorsed by the World Health Assembly, and can take credit for maintaining the momentum during the last decade, but ultimate success in controlling or eliminating NTDs will depend on overcoming the potent 21st century realities of resource constraints and social, ecological and environmental determinants of transmission^54,55^ in endemic countries to make the road map a reality.

## Data Availability

Not applicable.
